# Neuropathic and Psychogenic Components of Burning Mouth Syndrome: A Systematic Review

**DOI:** 10.3390/biom11081237

**Published:** 2021-08-18

**Authors:** Marie Orliaguet, Laurent Misery

**Affiliations:** 1 LIEN, Department of Oral Surgery, University of Western Brittany, F-29200 Brest, France; marie.orliaguet@chu-brest.fr; 2 LIEN, Department of Dermatology, University of Western Brittany, F-29200 Brest, France

**Keywords:** burning mouth syndrome, neuropathic, psychogenic, nociplastic pain

## Abstract

The pathophysiology of primary burning mouth syndrome (BMS) has been extensively debated but is poorly understood despite a large number of hypotheses attempting to explain its etiopathogenic mechanisms. The aim of the present work was to systematically review papers that could provide arguments in favour of the neuropathic and psychogenic components of primary BMS for a better understanding of the disease. This systematic review (SR) was registered in PROSPERO (CRD42021224160). The search was limited to articles in English or French from 1990 to 01 December 2020. A total of 113 articles were considered for data extraction. We divided them into four subgroups: pharmacological and nonpharmacological management studies (*n* = 23); neurophysiological studies (*n* = 35); biohistopathological studies (*n* = 25); and questionnaire-based studies (*n* = 30). Several of these studies have shown neuropathic involvement at various levels of the neuraxis in BMS with the contribution of quantitative sensory testing (QST), functional brain imaging, and biohistopathological or pharmacologic studies. On the other hand, the role of psychological factors in BMS has also been the focus of several studies and has shown a link with psychiatric disorders such as anxiety and/or depression symptoms. Depending on the patient, the neuropathic and psychogenic components may exist simultaneously, with a preponderance of one or the other, or exist individually. These two components cannot be dissociated to define BMS. Consequently, BMS may be considered nociplastic pain.

## 1. Introduction

The International Headache Society (IHS) defines burning mouth syndrome (BMS) as “an intraoral burning sensation for which no medical or dental cause can be found”. IHS diagnostic criteria include constant pain, a normal appearance of the oral mucosa, and exclusion of any local or systemic diseases [1]. The word “stomatodynia” is also sometimes used. Its prevalence has been reported to be between 0.01% and 40%, with the highest percentages in postmenopausal women and in older age ranges [2,3]. The burning sensation commonly involves the tip of the tongue and the lips and is often accompanied by xerostomia, taste disorders, or food hypersensitivity [4].

In 2003, Scala et al. proposed a distinction between “primary BMS”, an essential or idiopathic form without any organic cause, and “secondary BMS”, resulting from a local or systemic pathological condition [5]. The pathophysiology of primary BMS is extensively debated but poorly understood despite a large number of hypotheses attempting to explain its etiopathogenic mechanisms. Initially, studies often highlighted psychological causes [6,7], and primary BMS was initially classified as psychogenic pain [8]. In recent decades, it has been shown that several neuropathic mechanisms may contribute to the primary BMS pathophysiology. Indeed, neurophysiologic, psychophysical and functional imaging studies [9,10,11,12,13] have suggested pathophysiological alterations at different levels of the neuraxis, and BMS is currently considered to be a neuropathic pain affecting the central and peripheral nervous systems [1,14].

Some systematic reviews about BMS have already been published. Most of them evaluated the efficacy of pharmacological and/or nonpharmacological treatments [15,16,17,18,19,20,21,22]. A few others were interested in psychological factors [23], psychophysical characterization [24,25,26], or more specifically, cytokine levels and their role in the etiopathogenesis of BMS [27]. However, no systematic review has made an inventory of all available data on primary BMS to highlight the elements leaning more towards the neuropathic or psychogenic components or hypotheses.

The aim of the present work was to systematically review papers that could provide arguments in favour of the neuropathic and psychogenic components of primary BMS for a better understanding of the disease and its management.

## 2. Materials and Methods

### 2.1. Protocol Registration

This systematic review (SR) was registered in PROSPERO, the International Prospective Register of Systematic Reviews produced by the Center for Reviews and Dissemination of the University of York and funded by the National Institute for Health Research (NIHR) (registration number CRD42021224160—8 January 2021).

According to the population/exposure/comparator/outcome (PECO) pillars, we formulated the following question: to assess the neuropathic and psychogenic components (outcomes) of BMS in patients with primary BMS (exposure) [28].

### 2.2. Search Strategy

A computerized literature search for studies investigating different forms of BMS was performed on 1 December 2020 in the PubMed and Google Scholar databases using the following terms: “burning mouth syndrome” AND “pain”, “glossodynia” AND “pain” and “stomatodynia” AND “pain”. The search was limited to articles in English or French from 1990 to 1 December 2020.

### 2.3. Selection Criteria

The following types of papers were included: randomized clinical trial, control clinical trial, case–control study, cross-sectional study, cohort study, pathophysiological study, and systematic review with meta-analysis. Narrative reviews and case reports were not included. Articles were selected if they were related to pertinent arguments in favour of neuropathic and/or psychological components of primary BMS. Studies on antidepressants were not included because these drugs can be effective for both neurological and psychiatric disorders. Consequently, they cannot provide arguments to support any specific hypothesis.

The titles were screened for relevance and duplicates were removed. Abstracts were screened according to the PRISMA recommendations [29] by one of the authors (M.O.); a second author (L.M.) double-checked the article selection process. In cases of disagreement, M.O. and L.M. consulted each other to come to a consensus.

### 2.4. Data Extraction

The following data were extracted: article/study type, main outcomes/pertinent arguments in favour of neuropathic and/or psychological components, the results, and the risk of bias after reading all of the selected articles in full. All of the collected information was based on the focused question: a pertinent argument in favour of neuropathic or psychological components of primary BMS.

### 2.5. Data Analysis/Statistical Analysis

The data were collected according to the PRISMA checklist [29].

## 3. Results

### 3.1. Study Selection

**Figure 1:**
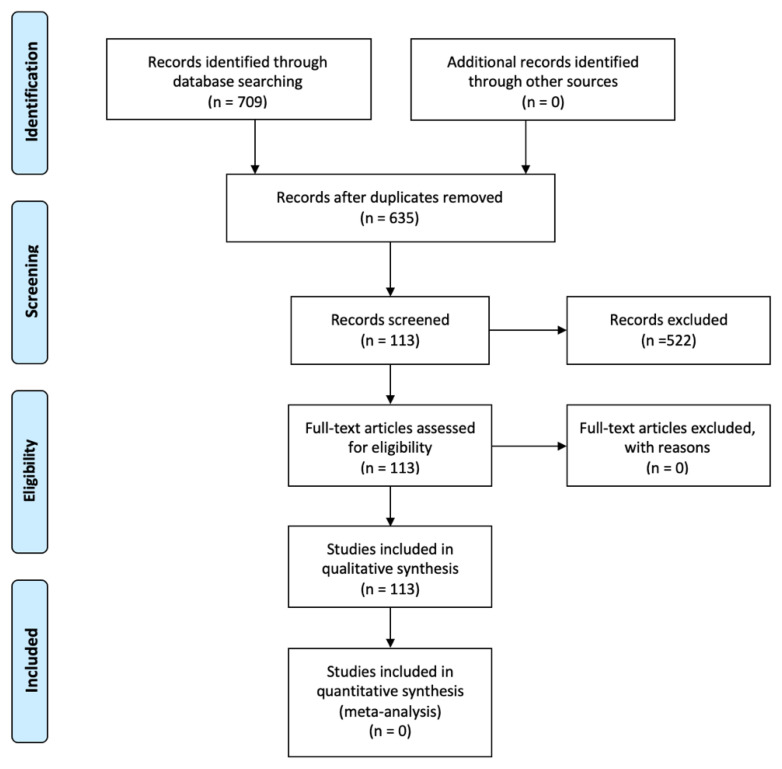
Prisma flow diagram of the systemic review process.

The study search process is summarized in the flow diagram (Figure 1).

The initial search found a total of 709 articles. After duplicates were removed and the records were screened, 113 articles were included for data extraction.

### 3.2. Study Characteristics

A total of 113 articles were considered for data extraction. To facilitate the analysis, we divided the 113 articles into four subgroups according to the subjects they dealt with: pharmacological and nonpharmacological management studies (*n* = 23); neurophysiological studies (*n* = 35); biohistopathological studies (*n* = 25); and questionnaire-based studies (*n* = 30).

### 3.3. Pharmacological and Non-Pharmacological Management Studies

Among the 23 studies, 1 was a meta-analysis [19], 16 were randomized clinical trials (RCTs) [30,31,32,33,34,35,36,37,38,39,40,41,42,43,44,45], and 6 were uncontrolled clinical trials [46,47,48,49,50,51].

In a meta-analysis, Häggman-Henrikson et al. [19] showed that clonazepam and capsaicin are effective for the treatment of BMS.

#### 3.3.1. Low Level Laser Therapy (LLLT)

Fourteen studies evaluated the effects of LLLT in reducing pain in BMS patients. Four uncontrolled clinical studies showed a significant improvement in BMS symptoms, ranging from 47.6% to 80.4% [46,47,48,49]. Among the 10 RCTs, 6 studies compared LLLT to placebo [31,33,35,37,38,39], 2 studies compared different LLLT techniques to each other and placebo [32,34], and 2 studies compared LLLT to other treatments [30,36].

Arduino et al. [36] compared an LLLT group to a clonazepam group. The laser group showed a significant improvement in pain parameters compared to the clonazepam group.

Barbosa et al. [30] compared an LLLT group to an alpha-lipoic acid (ALA) group. The results suggested that LLLT and ALA are both effective therapies for reducing burning mouth symptoms and that LLLT is more effective than ALA. Spanemberg et al. [33] showed that LLLT resulted in a significant reduction in pain compared to placebo. Sikora et al. [37] and De Pedro et al. [39] showed that LLLT resulted in a significant reduction in pain compared to the placebo group but it found no significant differences between the groups on the OHIP-14 (Oral Health Impact. Profile 14), while Arbabi-Kalati et al. [31], Bardellini et al. [35], Valenzuela et al. [34], and Spanemberg et al. [32] did. Pezelj-Ribaric et al. [38] found no significant difference in the improvement of pain between groups, but a decrease in the salivary levels of TNFα and IL-6 was seen after LLLT treatment.

#### 3.3.2. Alpha-Lipoic Acid (ALA)

Three articles evaluated the effect of ALA on pain in BMS patients. Cavalcanti et al. [40] and Carbone et al. [41] found no significant difference between the control and ALA groups. Femiano and Scully [42] found a significant improvement in the ALA group (97%) vs. the controls (40%) at 2 months.

#### 3.3.3. Capsaicin

Two articles evaluated the effect of capsaicin on pain in BMS patients. Petruzzi et al. [44] showed that patients treated with systemic capsaicin administered in 0.25% capsules twice a day were significantly better than the placebo group; the VAS score was significantly lower in treated patients (5.84 ± 1.17) than in the placebo-control group (6.24 ± 0.96). Jørgensen et al. [43], in a cross-over study, showed that treatment with a topical application of either 0.01% or 0.025% oral capsaicin gel on the dorsal part of the tongue three times daily significantly improved the burning symptoms, but there was no significant difference between the two concentrations of gels in relieving symptoms.

#### 3.3.4. Other Pharmacological Treatments

Valenzuela et al. [50] tried a 2% chamomile gel to treat patients with BMS and found no significant differences in pain compared with the placebo group.

Saraceno et al. [51] treated 15 patients with topical cyclosporine applied as a mouthwash for 4 weeks. Five out of 15 patients had a marked improvement, 6 patients showed a moderate response, 3 patients had a slight improvement, and 1 patient did not show any change. The cyclosporine mouthwashes appeared to be safe and beneficial for reducing the burning sensation in patients with BMS.

Formaker et al. [45] tested the effect of a topical anaesthetic (dyclonine HCl) on the patients’ intensity ratings for oral burning in a BMS cohort. For 12 patients, the burning increased, for 14 patients the burning did not change, and for 7 patients the burning decreased.

### 3.4. Neurophysiological Studies

#### 3.4.1. Quantitative Sensory Testing (QST)

Twelve articles explored the psychophysical characteristics of BMS. We found 2 systematic reviews with meta-analyses [24,26] and 10 clinical trials [52,53,54,55,56,57,58,59,60,61].

The QST outcomes were thermal detection thresholds, thermal pain thresholds, mechanical detection thresholds, and mechanical pain sensitivity. Warmth detection threshold (WDT), cold detection threshold (CDT), thermal sensory limen (TSL), paradoxical heat sensations (PHS), heat pain threshold (HPT), and cold pain threshold (CPT) were measured with special equipment that increased and decreased the temperatures. Mechanical detection (MDT) and pain thresholds (MPT) were evaluated using von Frey filaments and pinpricks applied with variable forces, following the “method of limits”. Additionally, other analysed parameters were mechanical pain sensitivity (MPS) using pinpricks, vibration detection thresholds (VDT) using stunning forks, and pressure pain threshold (PPT) by applying a pressure gauge tool.

In their meta-analysis, Madariaga et al. [24] showed significant differences between BMS patients and controls in warmth (effect size = 0.683; *p* < 0.05) and cold detection thresholds (effect size = −0.580; *p* < 0.001). Catley et al. [26] concluded that there was no difference between BMS patients and controls (from just one study for BMS).

Hartmann et al. [52] found loss of function in CDT, WDT, and MPT at the tongue and a gain of function in CPT at the tongue. Yilmaz et al. [53] found higher CDT and CPT and lower WDT and that some patients presented with allodynia, hyperalgesia, and/or hypoalgesia. Honda et al. [54] found no differences for tactile detection thresholds or filament-prick pain detection thresholds. Kaplan et al. [61] found no difference for WDT, CDT, CPT, and HPT, while they found that age was associated with an increase in WDT and a decrease in CDT, but not BMS. Forssell et al. [55] found 11 patients with a higher WDT, 4 patients with a higher CDT, and 18 patients with both thresholds increased. Kolkka et al. [56] found higher WDT and CDT. Mo et al. [57] found a lower CDT in the lower lip and the tongue, a higher WDT in the tongue, and a higher HPT in the tongue and lip, but no mechanical differences were found. Watanabe et al. [58] found a gain of function for MPS at the forearm and the tongue and a loss of function for MDT at the tongue. Yang et al. [59] found a higher WDT and a higher TSL but no mechanical differences. Moura et al. [60] found a higher PPT and a lower VDT.

#### 3.4.2. Other Neurophysiological Studies

In this sub-group, we included twelve articles.

Two clinical trials focused on a frequency analysis of heart rate variability (HRV) to explore autonomic dysfunction. Momota et al. [62] investigated whether the frequency analysis of HRV could reveal autonomic abnormalities associated with BMS and whether the autonomic changes were corrected after stellate ganglion near-infrared irradiation (SGR). Frequency analysis of HRV revealed an autonomic instability associated with BMS and enabled tracing of the autonomic changes corrected with SGR. Koszewicz et al. [63] performed HRV, deep breathing (exhalation/inspiration [E/I] ratio), and sympathetic skin response (SSR) tests to explore the autonomic nervous system in BMS patients and Parkinson’s disease patients compared to healthy subjects. The mean HRV and E/I ratios were significantly lower in the BMS and Parkinson disease groups. A significant prolongation of SSR latency in the foot was revealed in both the BMS and Parkinson disease patients, and a reduction of the SSR amplitude occurred in only the Parkinson disease group. The autonomic questionnaire score was significantly higher in the BMS and Parkinson disease patients than in the control subjects, with the Parkinson disease group having the highest scores.

Grémeau-Richard et al. [64] investigated the effects of a lingual nerve block on spontaneous burning pain and identified two groups of patients: a “peripheral group”, where the VAS score decreased due to lingual nerve injection of lidocaine or saline (*p* = 0.02), and a “central group”, where the pain intensity score increased after lidocaine injection and decreased after a saline injection.

Braud et al. [65] evaluated the taste function by electrogustometry in BMS subjects and healthy controls. The mean electrogustometric thresholds (EGMt) were significantly increased in patients with BMS on the right side of the dorsum of the tongue and on the right lateral side of the tongue. In the BMS group, the VAS scores were significantly correlated with EGMt at the tip of the tongue and on the right and left lateral sides of the tongue.

Jääskeläinen et al. [12] recorded the eye blink reflex (BR) in the BMS patients and the healthy control subjects. The BMS patients had significantly higher stimulus thresholds for the tactile R1 (tactile A-fibres, ipsilateral early component) components of the BR than the control subjects. With non-noxious stimulation, the BMS patients more frequently showed pain-related R3 (thin myelinated Ad-fibres) components than the controls. In addition, four BMS patients had abnormal habituation of the R2 (tactile A-fibres, ipsi-and contralateral late components) components.

Bonenfant et al. [66], through a self-administered, custom-made survey, determined the prevalence and characteristics of BMS in a Parkinson’s disease (PD) population. They found that the prevalence of BMS in PD was 4.0%; the PD severity and levodopa equivalent daily dose did not differ between the non-BMS and BMS participants but the mean poor oral health index was higher in the BMS compared to the non-BMS subjects.

De Siqueira et al. [67] investigated the sensory characteristics of orofacial pain in patients compared with control subjects by evaluating the thermal (cold and warm), tactile, and pain thresholds. They found that BMS patients had a loss of thermal perception and a decrease in tactile perception compared with the control subjects (*p* < 0.001).

Svensson et al. [68] used argon laser stimulation and showed that the sensory thresholds were significantly higher and the ratios between pain and sensory thresholds were significantly lower in patients with BMS. The pain thresholds were significantly elevated on the lower lip skin, the anterior hard palate, and the hand in patients with BMS.

Ito et al. [69] examined the pain threshold and pain recovery and found no significant differences between patients and controls in terms of the pain threshold on the finger. The threshold on the tongue was significantly higher in patients than in controls. Among the three types of stimulation (warm, cold, and mechanical), BMS patients perceived significantly higher pain from mechanical stimulation for the first 5 min after stimulation.

Siviero et al. [70] determined tactile, pain, thermal, gustative, and olfactory thresholds in a group of patients with BMS compared with controls. Somatosensory findings showed that BMS patients had higher tactile thresholds at the maxillary branch (*p* = 0.001) and higher warm thresholds at the maxillary (*p* = 0.032) and mandibular (*p* = 0.001) branches and that BMS patients had higher pain thresholds at the ophthalmic and maxillary branches (*p* = 0.05).

The gustative evaluation showed significant differences in all basic tastes (sweet *p* = 0.001, salty *p* = 0.004, sour *p* = 0.001, and bitter *p* = 0.001). The BMS patients had higher salty, sweet, and bitter thresholds but lower sour thresholds. The olfactory evaluation showed that BMS patients had higher olfactory thresholds.

Gao et al. [71] stimulated the dorsal linguae of BMS and control patients by electroneuromyography with pain and numbness to measure the pain threshold and the assessment of trigeminal somatosensory evoked potentials (TSEPs). The pain thresholds were significantly lower, and the spike potential appeared earlier in the BMS with pain group (*p* < 0.01).

Honda et al. [72] examined reports of perceptual distortion evoked by transient deafferentation and burning pain as models of aspects of BMS. Healthy women took part in three experimental sessions that included exposure to a lingual nerve block, capsaicin, and a control substance. There was a significantly higher MDT on the tongue during the lingual nerve block session at 5 min up until 1 h, with the perceived tongue size significantly increased at 5, 15, and 30 min and at 1 h compared to the baseline (*p* < 0.05). Although, the perceived size determined by the Numerical Rating Scale scores during the capsaicin session was significantly larger for the lower lip at 5 min compared to baseline (*p* < 0.001), while there were no significant effects on the MDT or the perceived sizes for the tongue, lower front teeth, or right thumb at any of the time points.

#### 3.4.3. Functional Brain Imaging

Eleven studies used functional magnetic resonance imaging (fMRI) to explore cerebral activation or structure in BMS patients.

Albuquerque et al. [9] showed less volumetric activation in the entire brain to painful hot stimuli in primary BMS patients compared to healthy control subjects and, more specifically, in the bilateral thalamus. Nevertheless, BMS patients displayed greater fractional signal changes in the right anterior cingulate cortex (BA 32/24) and bilateral precuneus than controls.

Tan et al. [73] explored the brain anatomical and functional changes in BMS and showed lower grey matter volume (GMV) in the bilateral ventromedial prefrontal cortex (VMPFC) and increased functional connectivity between this region and the bilateral amygdala.

Zavoreo et al. [74] showed that there was a significant difference in echogenicity of the substantia nigra and midbrain raphe (hypoechogenicity) and brain nucleus (hyperechogenicity) in patients with BMS as compared to the controls.

Liu et al. [75] showed that patients with BMS displayed significantly different depression and anxiety scales compared to the control group, and significantly lower regional cerebral blood flow in the left parietal and left temporal lobes was recorded for BMS patients with depression.

Khan et al. [76] investigated the brain grey matter volume (GMV) with voxel-based morphometry (VBM), white matter fractional anisotropy (FA) with diffusion tensor imaging (DTI), and functional connectivity in resting-state functional MRI (rsfMRI). Compared to healthy controls, BMS patients had increased GMV and lower FA in the hippocampus (Hc) and decreased GMV in the medial prefrontal cortex (mPFC). rsfMRI revealed altered connectivity patterns during different states of pain/burning, with increased connectivity between the mPFC (a node in the default mode network) and the anterior cingulate cortex, occipital cortex, ventromedial PFC, and bilateral Hc/amygdala in the afternoon compared with the morning session.

Yoshino et al. [77] examined the activation of brain regions in response to intraoral tactile stimuli when modulated by angry facial expressions. Compared to healthy controls, BMS patients exhibited higher tactile ratings and greater activation in the postcentral gyrus during the presentation of tactile stimuli involving angry faces relative to controls and a significant positive correlation between changes in tactile-related activation of the postcentral gyrus elicited by angry facial expressions and pain intensity in daily life.

Sinding et al. [78] analysed the grey matter concentration to highlight changes in the central system of subjects with BMS in a retrospective analysis. They found that a major part of the ‘pain matrix’ presented modifications of the grey matter concentration in subjects with BMS. Six regions out of eight were affected (anterior and posterior cingulate gyrus, lobules of the cerebellum, insula/frontal operculum, inferior temporal area, primary motor cortex, and dorsolateral prefrontal cortex (DLPFC)). In the anterior cingulate gyrus, the lobules of the cerebellum, the inferior temporal lobe, and the DLPFC, the pain intensity correlated with the grey matter concentration.

Lee et al. [79] showed that the grey matter volume was reduced in the left thalamus and left middle temporal gyrus in the BMS group than in the controls. The regional cerebral blood flow in the BMS group was significantly decreased in the left middle temporal gyrus, left insula, right middle temporal gyrus, and right insula compared with the controls. In the BMS patients, there was a significant correlation between the GMV and pain severity in the left middle temporal gyrus.

Wada et al. [80] investigated the brain network of the BMS brain by using probabilistic tractography and graph analysis and showed that in the BMS brain, a significant difference in local brain connectivity was recognized in the bilateral rostral anterior cingulate cortex, right medial orbitofrontal cortex, and left pars orbitalis, which are parts of the medial pain system.

Hagelberg et al. [81] focused on striatal dopamine D1 and D2 receptors in BMS by using 11C-NNC 756 and 11C-raclopride binding in a PET study. The striatal uptake of 11C-NNC 756 did not differ between patients and controls. In a voxel-level analysis, the uptake of 11C-raclopride was significantly higher in the left putamen in burning mouth patients (corrected *p*-value of 0.038 at the cluster level). In the region of interest analysis, the D1/D2 ratio was 7.7% lower in the right putamen and 6.4% lower in the left putamen than in the controls.

Shinozaki et al. [82] compared the brain response to noxious heat stimuli in right-handed women with primary BMS and sex- and age-matched right-handed healthy female controls. Patients and controls both reported feeling more pain during palm stimulation than during lip stimulation. Repetition of noxious heat stimulus on the lower lip but not on the palm induced habituation in brain activity in the cingulate cortex without a reduction in pain perception. Multiple regression analysis revealed a correlation between the perceived pain intensity and a suppression of brain activity in the anterior cingulate cortex when the repeated thermal sequence was applied to the lower lip.

### 3.5. Bio-Histopathological Study

#### 3.5.1. Cytokine Levels

Among the six articles, five were case-control studies [83,84,85,86,87] and one was an RCT [88] (Table 1).

Ji et al. [84] evaluated IL-18 in saliva and found that IL-18 was increased in BMS compared to the control.

Treldal et al. [88] found that the plasma levels of IL-6, IL-8, IL-17, IL-23, and TNF-α were higher in BMS patients who were not given a local anaesthetic than in those who were, but without significant differences (*p*-values: 0.068–0.916). Only IL-6 was significantly elevated in the stimulated parotid saliva for the no effect group (*p* = 0.020). The cytokine levels in chewing-stimulated whole saliva did not differ between the patient groups.

Barry et al. [83] evaluated the plasma protein levels of Th1 cytokines (IFN-c, IL-2, IL-12p70, and TNF-a), Th2 cytokines (IL-4, IL-10, IL-6, and IL-13), and the chemokine IL-8 and found that IL-8 was increased in BMS patients compared to the controls.

Chen et al. [85] examined alterations in serum interleukin-6 and its clinical significance in BMS patients. Serum interleukin-6 in BMS patients was much lower than that in the controls and it was negatively correlated with the VAS values (*p* = 0.011).

Miyauchi et al. [86] evaluated the plasma levels of 28 neuroinflammation-related molecules in controls and BMS patients both pretreatment and 12-week post-treatment with duloxetine.

IL-1β, IL-1 receptor antagonist, IL-6, macrophage inflammatory protein-1β, and platelet-derived growth factor-bb were significantly higher in BMS patients than in the controls. Guimarães et al. [87] evaluated the interleukin-1β and serotonin transporter gene (5-HTTLPR) polymorphisms in BMS patients and found no significant difference in 5-HTTLPR genotypes between the case and control groups (*p* = 0.60); however, a significant increase was observed in the IL-1β high production genotype CT in BMS subjects (*p* < 0.005).

#### 3.5.2. Histological Studies

Eight studies were focused on histological analyses; six of them were performed on tongue biopsies [11,13,89,90,91,92], one used exfoliative cytology of the oral mucosa [93], and the final one was performed with corneal confocal microscopy [94].

The analysis of the tongue biopsies showed that patients with BMS had significantly lower intraepidermal [90] and epithelial [11,13,89] nerve fibre density than healthy subjects.

Beneng et al. published two studies. One aimed to study Nav1.7 (sodium channel) expression in dental pulpitis pain, an inflammatory condition, and in BMS [91]; they showed that Nav1.7 immunoreactive fibres were seen in abundance in the submucosal layer of the tongue biopsies, with no significant difference between the BMS patients and controls, and that there was a significantly increased visual intensity score for Nav1.7 in nerve fibres in painful dental pulp specimens compared to the controls. Another study was about sensory purinergic receptor P2X3 immunoreactivity levels in the lingual mucosa in BMS patients [92]. P2X3-positive fibres were significantly increased in BMS compared with the controls (*p* = 0.024). In contrast, neurofilament-stained fibres were reduced in BMS, and when expressed as a ratio of the neurofilament percentage area, there was a trend for an increase in P2X3-positive fibres in the BMS group.

Wandeur et al. [93] revealed that oral epithelial cells from smears of BMS patients exhibited significant cytomorphometric changes, with a significant predominance of nucleated cells of the superficial layer compared with healthy controls (*p* = 0.00001).

O’Neil et al. [94] used corneal confocal microscopy to show that the corneal nerve fibre density and corneal nerve fibre length were significantly lower in BMS patients than in the controls.

#### 3.5.3. Other Biological Studies

Two studies explored the presence of mineral elements and vitamins in BMS patients.

Morr Verenzuela et al. [95] found that, among 659 patients with BMS, the most common decreased values or deficiencies blood levels were those of vitamin D3 (15% of patients), vitamin B2 (15%), vitamin B6 (5.7%), zinc (5.7%), vitamin B1 (5.3%), thyrotropin (TSH) (3.2%), vitamin B12 (0.8%), and folic acid (0.7%) and that laboratory values for fasting blood glucose and TSH were increased by 23.7% and 5.2%, respectively, in their retrospective analysis of BMS patients.

López-Jornet et al. [96] did not find significant differences in mineral and trace metals in unstimulated whole saliva.

Boucher et al. [97] found higher blood opiorphin levels in BMS patients than in controls.

Barry et al. [98] measured endocannabinoid (anandamide (AEA) and 2-arachidonoyl-glycerol (2-AG)) ligands and non-cannabinoid *N*-acylethanolamine (palmitoylethanolamide (PEA) and oleoylethanolamide (OEA)) molecules in the plasma of BMS patients and healthy controls. They found that plasma levels of PEA, but not OEA, AEA, or 2-AG, were significantly higher in patients with BMS and that plasma PEA, OEA, and AEA levels were correlated with depressive symptomatology.

Tatullo et al. [99] aimed to assess the relationship between oxidative stress and BMS in female patients and found that women with BMS had significantly different d-ROM (total oxidant capacity) and BAP (biological antioxidant potential as iron-reducing activity) levels.

Kang et al. [100] found that salivary progesterone levels had significant positive correlations with oral mucosal epithelial MUC1 expression levels and with salivary cortisol and DHEA (dehydroepiandrosterone) levels. The salivary level of 17b-oestradiol showed significant positive correlations with the symptom duration, the severity of the effects of oral complaints on daily life, and the results from psychological evaluations. The cortisol level in unstimulated whole saliva (UWS) and the cortisol/DHEA ratio in UWS and stimulated whole saliva had significant negative correlations with the severity of the oral burning sensation. The severity of the taste disturbance had significantly positive correlations with the results from psychometry.

Loeb et al. [101] reported that the saliva-buffering capacity and the contents of protein and hyaluronic (HA) acid were similar between the BMS and control groups. In contrast, the chondroitin sulphate (CS) concentration was decreased in the saliva of patients with glossodynia compared to the control group (*p* = 0.0036). On the other hand, glandular kallikrein had increased activity in the saliva of patients compared to normal subjects (*p* < 0.0001).

Koike et al. [102] examined the role of immune and endocrine functions in the pathology of BMS. They found that BMS patients were significantly more anxious than the controls (*p* = 0.011) and that the plasma adrenaline levels were significantly lower (*p* = 0.020) in BMS patients than in the controls. Linear regression analysis of all patients combined revealed that depression levels were significantly positively associated with plasma noradrenaline and cortisol levels (*p* = 0.002 and 0.001, respectively). However, compared with controls, BMS patients had a significantly lower CD8(+) cell count (*p* < 0.001) and a significantly higher CD4/CD8 ratio (*p* = 0.002). Discriminant analysis revealed that the CD8(+) cell count and CD4/CD8 ratio were independent variables that distinguished BMS patients from the controls.

Heckmann et al. [103] studied oral mucosal blood flow in BMS patients using laser Doppler flowmetry. Measurements were made at rest and over 2 min following dry ice application for a 10 s duration using a pencil-shaped apparatus. When compared to healthy controls, BMS patients generally exhibited larger changes in mucosal blood flow, and those changes were significant for recordings made on the hard palate. In general, vasoreactivity in BMS patients was higher than that in healthy controls.

Zidverc-Trajkovic et al. [104] measured the levels of calcitonin gene-related peptide (CGRP) in the saliva of BMS patients and showed that the levels of CGRP were nonsignificantly decreased in BMS patients in comparison to healthy subjects.

Shinoda et al. [105] assessed the mRNA expression of Artn in the tongue mucosa of patients with burning mouth syndrome. There was no significant difference in the health history between patients with BMS and the control subjects. The mRNA expression of Artn in the tongue mucosa of patients with BMS was significantly higher than that of the control subjects.

### 3.6. Questionnaire-Based Studies

We found 30 articles that studied the involvement of psychological factors in BMS (Table 2).

Galli et al. [23] performed a systemic review and meta-analysis on this topic and found that all studies but one reported at least some evidence for the involvement of psychological factors in BMS. Anxiety and depression were the most common and most frequently studied psychopathological disorders among BMS patients.

The majority of studies searched for the presence of psychiatric disorders, with a main focus on depression and/or anxiety, using several different scales and questionnaires. Four of them used the Hospital Anxiety and Depression Scale (HADS) [106,107,108,109]; Lopez-Jornet et al. [106] found a higher level of anxiety (*p* = 0.008) and depression (*p* = 0.005) with the HADS, as did Malik et al. [107]. Four studies used the SCL-90-R (Symptom Checklist-90-Revised) [6,110,111,112]; Eli et al., with the SCL-90-R, found a relatively high psychopathologic profile, especially for the scales of somatization and depression, and found significant correlations between the intensity of pain experienced by the patients and some of the SCL-90 scales (somatization, depression, anxiety, GSI, and PSDI). Three studies used the Hamilton Rating Scale for Depression and Anxiety (HAM-D, HAM-A) [111,113,114], and three used the Beck Depression Inventory (BDI) and/or the Beck Anxiety Inventory (BAI) [115,116,117]; Tokura et al. [117] found a higher depression level with BDI, as did Das Neves de Araújo Lima et al. [115] (*p* = 0.033). Three studies used Zung’s Self-Rating Depression Scale (SRSD) [7,118,119,120], and two used the State-Trait Anxiety Inventory (STAI) [7,111]. Bogetto et al. used the BPRS (Brief Psychiatric Rating Scale) [121] and found that the frequency of psychiatric disorders was significantly higher in BMS subjects in both the current and lifetime perspectives (*p* < 0.001). In their study, Forssell et al. [122] used the DEPS (self-rating Depression Scale) and the PASS-20 (Pain Anxiety Symptom Scale-20) and found that patients in the highest intensity and interference tertiles reported more depression (*p* = 0.0247 and *p* = 0.0169) and pain/anxiety symptoms (*p* = 0.0359 and *p* = 0.0293) and were more preoccupied with pain (*p* = 0.0004 and *p* = 0.0003) than patients in the low intensity and interference groups. Schiavone et al. [111], who used the SCL-90-R, STAI, and HAM-D questionnaires, found higher scores of anxiety (STAI Y1, *p* = 0.026 and STAI Y2, *p* = 0.046), higher scores of depression (*p* < 0.001), higher SCL-90-R scores of somatization (*p* = 0.036), hostility dimensions (*p* = 0.028), and that their pain was affected by depression (*p* < 0.005), and that depression was affected by anxiety (*p* < 0.001).

Three studies explored alexithymic traits with the Toronto Alexithymic Scale questionnaire (TAS) [114,116,123]. Miyaoka et al. [123] found that alexithymia was significantly higher in BMS patients. Marino et al. [114] also showed that alexithymia was significantly higher in BMS patients (79.3% vs. 6.9%; *p* < 0.001) and that alexithymia did not show any correlation with anxiety and depression. In a study by Jerlang et al. [116], only a weak tendency was seen between alexithymic traits and depressive symptoms (*p* = 0.0577), and no correlation was found between alexithymic traits and pain, well-being, depression or anxiety.

Regarding oral quality of life, three studies used the OHIP-14 (Oral Health Impact Profile-14) [106,113,124] and one used the GOHAI (Geriatric Oral Health Assessment Index) [113]. Lopez-Jornet et al. [106] found no significant result for the quality of life, while Acharya et al. [124] found that the OHIP-14 was significantly worse in BMS patients than in the control group. Adamo et al. [113] showed that the scores from all outcome measurements were significantly different between the cases and controls (*p* < 0.001), with a strong correlation between the GOHAI and the OHIP-14 (*p* < 0.001).

Two studies explored sleep quality and used the PSQI (Pittsburgh Sleep Quality Index) and the EES (Epworth Sleepiness Scale) [106,119]. Lopez-Jornet et al. [106] found a worse sleep quality in BMS patients than in control subjects (*p* ≤ 0.001) and identified anxiety and depression as significant determinants of poor sleep quality. Tu et al. [119] showed that patients with comorbid psychiatric disorders had a higher rate of sleep disturbance.

Regarding personalities, Taiminen et al. [125] used the Structured Clinical Interview for DSM-IV (SCID) to explore psychiatric and personality disorders and found no differences in the rates of psychiatric and personality disorders between BMS and atypical facial pain patients. Merigo et al. [126] used the MMPI-II (Minnesota Multiphasic Personality Inventory) and found no significant differences for the MMPI-II scales. Tokura et al. [117] used the TCI (Temperament and Character Inventory) and found significantly lower novelty seeking (*p* = 0.009) and self-directedness (*p* = 0.039) and significantly higher harm avoidance (*p* < 0.001).

Jedel et al. [127] used the SSP (Swedish Universities Scales of Personality); the SSP subscales of somatic trait anxiety, psychic trait anxiety, stress susceptibility, and verbal trait aggression differed between women with BMS and controls, and the personality factor scores for neuroticism and aggressiveness were higher. Komiyama et al. [128] used the RDC-TMD (Research Diagnostic Criteria for Temporomandibular Disorders) to explore their psychosocial characteristics and found significantly higher somatization scores (*p* = 0.027) and significantly higher depression scores (*p* = 0.001).

Some studies explored the neuropathic component. Heo et al. [129] evaluated and compared the validity of the PainDETECT, DN4 (Douleur Neuropathique 4 questionnaire), and the abbreviated DN4 (DN4i) neuropathic pain questionnaires for primary BMS. The mean areas under the ROC curves (AUCs) for PainDETECT, DN4, and DN4i were 0.81, 0.79, and 0.81, respectively. There were no statistically significant differences in the AUCs among the questionnaires. The total scores for PainDETECT, DN4, and DN4i in the primary BMS group were significantly associated with the pain intensity. Sevrain et al. [108] evaluated the neuropathic and psychological components of the BMS with the DN4i and HADS questionnaire. Thirty-one percent of patients with BMS had a DN4i score in favour of neuropathic pain, and 34.3% had a HADS overall score in favour of anxiety and depressive disorder. Lopez-Jornet et al. [109] used the PainDETECT questionnaire, and 21% of BMS patients had total scores >19, indicating the presence of neuropathic pain. Braud et al. [130] used the DN4 questionnaire; the DN4 scores ranged from 2 to 7 (mean score: 3.9 ± 0.3), and 59% of the patients had a DN4 score ≥ 4. These findings support the use of DN4 as a tool for screening for BMS and reinforced the view that BMS is a clinical manifestation of a neuropathic disease.

Zakrzewska et al. [131] used pain cards (photographic images co-created by an artist and chronic pain patients) in groups of BMS patients to characterize their pain and its impact on their quality of life. They found that the choice of pain card and the words used to explain the choice implied a neuropathic type of pain. Themes that were common included those of isolation, loss of confidence, low mood, and a decrease in activities and socialization.

In their controlled clinical study, Mignogna et al. [132] worked on unexplained somatic comorbidities in patients with BMS. Oral symptoms were collected by a specialist in oral medicine and a general dentist, while data concerning unexplained extraoral symptoms were gathered by each specialist ward, i.e., ophthalmology, gynaecology, otolaryngology, gastroenterology, neurology, cardiology, internal medicine, and dermatology. In the BMS group, 96.1% of patients reported unexplained extraoral symptoms, while 3.9% of patients reported only oral symptoms. Painful symptomatology in different bodily regions was reported more frequently by BMS patients (83.3%) than by oral lichen planus (OLP) patients (1.8%) and healthy patients (11.7%) (*p* < 0.001). The differences in the overall unexplained extraoral symptoms between BMS (96.1%) and OLP patients (9.3%) (*p* < 0.001) and between BMS (96.1%) and healthy patients (15.7%) (*p* < 0.001) were statistically significant.

**Table 2:** Overview of the included questionnaire-based studies (in alphabetic order by author).

Author/Year	Questionnaire(s)	Significant Results
Acharya, 2018 [124]	OHIP-14 (Oral Health Impact Profile-14)	OHIP 14 significantly poorer for the BMS group (*p* < 0.001)
Adamo, 2020 [113]	GOHAI (Geriatric Oral Health Assessment Index) OHIP-14 (Oral Health Impact Profile-14) VAS (Visual Analogue Scale) HAM-D and HAM-A (Hamilton Rating Scales for Depression and Anxiety)	Scores from all outcome measurements statistically different between the cases and controls (*p* < 0.001) with a strong correlation between the GOHAI and the OHIP-14 (*p* < 0.001). BMS patients: significant improvement in the VAS, HAM-D and HAM-A scores from time 0 to time 1 (*p* < 0.001), and in the OHIP-14 scores (*p* < 0.004) after the psychotropic drugs treatment
Bogetto, 1998 [121]	BPRS (Brief Psychiatric Rating Scale)	Higher mean BPRS total score (*p* < 0.005) Higher psychiatric disorders frequency (*p* < 0.001)
Braud, 2013 [130]	DN4 questionnaire VAS (Visual Analogue Scale)	DN4 scores ranged from 2 to 7 (mean score: 3.9 ± 0.3) 59% of the patients: DN4 score ≥ 4. The findings support the use of DN4 as a tool for screening BMS and reinforce the view that BMS is a clinical manifestation of a neuropathic disease
Carlson, 2000 [112]	McGill Pain Questionnaire MPI (Multidimensional Pain Inventory) SCL-90R (Symptom Checklist-90-Revised)	Patients did not report significant psychologic distress. Individual cases (7 of 33, or 21%): psychometric data indicated a likelihood of psychologic distress.
das Neves de Araújo Lima, 2016 [115]	Lipp’s Inventory of Stress Symptoms for Adults Beck Depression and Anxiety Inventory	Significant differences between groups, which were more prevalent in the BMS group: —in the presence of xerostomia (*p* = 0.01), —hyposalivation at rest (*p* < 0.001), —symptoms of depression (*p* = 0.033).
Eli, 1994 [6]	SCL-90 R questionnaire (General Symptomatic Index (GSI), Positive Symptom Total (PST), and Positive Symptom Distress Index (PSDI))	—Higher psychopathologic profile, especially on the scales of somatization and depression. —Significant correlations between the intensity of pain experienced by the patients and some of the SCL-90 scales (somatization, depression, anxiety, GSI, and PSDI).
Forssell, 2020 [122]	DEPS (Self-rating Depression Scale) PASS-20 (Pain Anxiety Symptom Scale-20) PVAQ (Pain Vigilance and Awareness Questionnaire)	Patients were divided into groups based on pain severity distribution tertiles: low intensity (NRS ≤ 3.7) or interference (NRS ≤ 2.9) (tertiles 1–2, *n* = 35) and moderate to intense intensity (NRS > 3.7) or interference (>2.9) (tertile 3, *n* = 17). Patients in the highest intensity and interference tertiles reported more depression (*p* = 0.0247 and *p* = 0.0169) and pain anxiety symptoms (*p* = 0.0359 and *p* = 0.0293), and were more preoccupied with pain (*p* = 0.0004 and *p* = 0.0003) than patients in the low intensity and interference groups. The score of the pain vigilance questionnaire correlated significantly with pain intensity (*p* = 0.009) and interference (*p* = 0.009). Depression (*p* = 0.003) and pain anxiety symptoms (*p* = 0.001) correlated with pain interference.
Galli, 2017 [23]	Meta-Analysis	All studies but one reported at least some evidence for the involvement of psychological factors in BMS. Anxiety and depression were the most common and the most frequently studied psychopathological disorders among BMS patients.
Heo, 2015 [129]	PainDETECT (neuropathic pain questionnaire) DN4, and abbreviated DN4 (DN4i)	Total scores for PainDETECT, DN4, and DN4i in the primary BMS group were significantly associated with pain intensity.
Jedel, 2020 [127]	SSP (Swedish universities scales of personality) PSQ (Perceived stress questionnaire)	SSP: subscales Somatic Trait Anxiety, Psychic Trait Anxiety, Stress Susceptibility and Verbal Trait Aggression differed between BMS and controls and the personality factor scores for Neuroticism and Aggressiveness were higher. PSQ: Higher perceived stress.
Jerlang, 1997 [116]	Beck’s depression inventory of 42 items Spielberger’s State-Trait Anxiety Scale TAS-20 (Toronto Alexithymic scale questionnaire)	Significant correlations between disability and alexithymic traits (*p* < 0.05), depressive traits (*p* < 0.001), state-anxiety (*p* < 0.05), trait-anxiety (*p* < 0.05) and VAS well-being (*p* < 0.01).
Kim, 2018 [110]	SCL-90-R (Symptom Checklist-90-Revised)	Patients with psychological problems: —Higher mean age, reduced stimulated whole saliva flow rate, and lower level of education than those without psychological problems —Higher rates and greater severity of various types of BMS-related symptom in most parts of the oral mucosa, higher rates of stress-related symptoms, and greater difficulties in daily activities Psychological problems in BMS patients are associated with an aggravation of BMS symptoms
Komiyama, 2012 [128]	Research Diagnostic Criteria for Temporomandibular Disorders	Chronic BMS patients: —Higher somatization score than those with acute BMS (*p* = 0.027) —Higher depression scores than those with acute BMS (*p* = 0.001).
Lopez-Jornet, 2015 [106]	HADS (Hospital Anxiety and Depression Scale) OHIP-14 (Oral Health Impact Profile-14) PSQI (Pittsburgh Sleep quality index) EES (Epworth Sleepiness Scale)	HADS: Higher level of anxiety (*p* = 0.008) and depression (*p* = 0.005) OHIP-14: no significant result Higher poor sleep quality in BMS patients (*p* ≤ 0.001). Anxiety and depression were identified as significant determinants of poor sleep quality
Lopez-Jornet, 2017 [109]	PainDETECT (neuropathic pain questionnaire) HADS (Hospital Anxiety and Depression Scale), VAS (Visual Analogue Scale)	Pain-VAS: Higher in BMS group PainDETECT obtained total scores >19 in 21% of BMS patients, indicating the presence of neuropathic pain Almost a third of BMS patients present neuropathic pain, which is strongly associated with the intensity of pain measured using VAS.
Malik, 2012 [107]	HADS (Hospital Anxiety and Depression Scale) GHQ-28 (General Health Questionnaire-28) VAS (Visual Analogue Scale)	Significant increase in the levels of anxiety and depression in the BMS group.
Marino, 2015 [114]	TAS-20 (Toronto Alexithymic scale questionnaire) HAM-A (Hamilton Rating Scales for Anxiety) Montgomery–Asberg Depression Rating Scale	79.3% of alexithymic patients (vs. 6.9%) (*p* < 0.001)
Merigo, 2007 [126]	MMPI-II (Minnesota Multiphasic Personality Inventory-2)	No significant differences on the MMPI-II scales
Mignogna, 2011 [132]	Oral symptoms collected by specialist in oral medicine and general dentist. Data concerning unexplained extraoral symptoms gathered by each specialist ward, i.e., ophthalmology, gynaecology, otolaryngology, gastroenterology, neurology, cardiology, internal medicine, and dermatology.	BMS group: —98 (96.1%) patients reported unexplained extraoral symptoms. —4 (3.9%) patients reported only oral symptoms. —Higher painful symptomatology in different bodily regions frequency (*p* < 0.0001). The differences in the overall unexplained extraoral symptoms between BMS (96.1%) and OLP patients (9.3%) (*p* < 0.0001) and between BMS (96.1%) and healthy patients (15.7%) (*p* < 0.0001) were statistically significant.
Miyaoka, 1996 [123]	EPQ (Eysenck Personality Questionnaire) TAS (Toronto Alexithymia Scale) GHQ (General Health Questionnaire)	EPQ: significantly lower on BMS patients TAS: significantly higher on BMS patients GHQ: no significant difference
Ott, 1992 [120]	Erlangen depression scale Scale for general somatic symptoms Anxiety-aggression scale Self-rating scale for state of wellbeing Self-rating depression scale	Psychiatrically relevant disorder, usually depression, for the majority of BMS patients
Schiavone, 2012 [111]	SCL-90-R (Symptom Checklist-90-Revised) STAI (State-Trait Anxiety Inventory Form Y 1–2) HAM-D (Hamilton Rating Scale for Depression)	—STAI: higher scores of anxiety (STAI Y1, *p* = 0.026 and STAI Y2, *p* = 0.046). —HAM-D: higher scores of depression (*p* < 0.001). —SCL-90-R: higher scores on somatization (*p* = 0.036) and hostility dimensions (*p* = 0.028). Pain is affected by depression (*p* < 0.005). Depression is affected by anxiety (*p* < 0.001).
Sevrain, 2016 [108]	DN4i HADS (Hospital Anxiety and Depression Scale) QDSA (French adaptation of the McGill Pain Questionnaire)	—31% had a DN4i score in favour of neuropathic pain. —34.3% had a HADS overall score in favour of anxiety and depressive disorder.
Taiminen, 2011 [125]	SCID-I and II (Structured Clinical Interview for DSM-IV)	No differences in the rates of psychiatric and personality disorders between BMS and AFP patients.
Takenoshita, 2010 [118]	ICD-10 (International Statistical Classification of Disease and Related Health Problems, Tenth Revision (F0–F9)) Zung’s Self-Rating Depression Scale SF-MPQ (Short-form Mcgill Pain Questionnaire) Present Pain Intensity (PPI) scale	The proportion of F4 classification (neurotic, stress-related, and somatoform disorders) in AO patients was significantly higher than in BMS patients. BMS patients were more frequently given a F3 classification (mood/affective disorders). 50.8% of BMS patients and 33.3% of AO patients had no specific psychiatric diagnoses. Depression rating: —21.4%: normal; —46.4%: neurotic tendency; —32.1%: depressive tendency.
Tokura, 2015 [117]	TCI (Temperament and Character Inventory) BDI (Beck Depression Inventory)	TCI: —Lower novelty seeking (NS) (*p* = 0.009); —Lower self-directedness (*p* = 0.039); —Higher harm avoidance (*p* < 0.001). BDI: Higher depression level
Trikkas, 1996 [7]	Zung’s Self-Rating Scale for Depression STAI (Spielberger’s State-Trait Anxiety Inventory) EPQ (Eysenck’s Personality Questionnaire) HDHQ (Fould’s Hostility and Direction of Hostility Questionnaire) SSPS (Schalling–Sifneos’ Personality Scale)	—Significantly higher values in the N (neuroticism) and the L (lie) factors of the EPQ. —Significantly higher rates of psychosomatic morbidity. —Significantly differentiated from controls with respect to all factors of HDHQ.
Tu, 2018 [119]	Short-Form McGill Pain Questionnaire (SF-MPQ) Zung Self-Rating Depression Scale	AO-BMS patients rated overall pain score and present pain intensity significantly higher than did the AO-only patients (*p* = 0.033 and *p* = 0.034). Patients having comorbid psychiatric disorders had a higher proportion of sleep disturbance in both groups and a higher proportion of depressive state in the AO-only group.
Zakrzewska, 2019 [131]	Visual Imagery investigate how photographic images (Pain Cards) co-created by an artist and chronic pain patients could be used in groups of patients with burning mouth syndrome to facilitate characterization of their pain and its impact on quality of life.	The choice of Pain Card and words used to explain the choice implied a neuropathic type of pain. Themes that were common included those of isolation, loss of confidence, low mood, and decrease in activities and socialization.

## 4. Discussion

### 4.1. Arguments for Neuropathic Pathogenesis

The peripheral nervous system seems to be implicated in BMS development, as shown by Laurie et al., Yilmaz et al., Penza et al. and Puhakka et al. [11,13,89,90], where the analysis of tongue biopsies showed that patients with BMS had a significantly lower intraepidermal [90] and epithelial [11,13,89] nerve fibre density than healthy subjects, suggesting small fibre neuropathies. Increases in the expression of nerve growth factor (NGF), TRPV1, and the purinergic P2X3 receptor in the remaining fibres have been shown in biopsies. Braud et al. [65] showed that the mean electrogustometric thresholds (EGMt) were significantly increased in patients with BMS; these abnormalities on electrogustatometry indicate the involvement of small Aδ taste afferents.

QST is a standardized method to characterize the somatosensory phenotype of patients with neuropathic pain and enables the study of central and peripheral sensitization. The use of this technique is highly recommended for the assessment of small- and large-fibre alterations after considering other parameters, such as the pain level and the neurological and psychological status of the patients [133]. However, according to previous studies [52,53,54,55,56,57,59,60,61], the results are contradictory.

Some other studies highlighted peripheral nervous system involvement in BMS. Brain stem reflex recordings (masseter reflex, blink reflex) are useful for the examination of the trigeminofacial system. Those tests revealed distinct abnormalities within the trigeminofacial large fibre system and its central connections. Some BMS patients had evident trigeminal system lesions that may be located within the peripheral nerves (lingual nerve, mandibular nerve, or the entire trigeminal nerve) or within the brainstem. Nervous system lesions are most often unilateral with bilateral spread of burning pain symptoms over time. Some other BMS patients showed signs of decreased inhibition of the blink reflex in the form of absent or deficient habituation of the reflex when stimulating the supraorbital nerve distribution outside the symptomatic intraoral trigeminal area [12,55,90].

fMRI studies have explored the implications of the central nervous system and support the hypothesis of a neuropathic aetiology of BMS; indeed, patients with BMS display structural and functional deficits in key brain regions associated with pain perception [9,73,75,76,78,80,81] and they frequently show morphofunctional changes, suggesting a neurogenic basis of BMS, but some also show markers of depression.

Moreover, some authors explored the neuropathic component with studies based on neuropathic pain questionnaires (PainDETECT, DN4, and DN4i) and found arguments in favour of neuropathic pain.

Controversial results were obtained for cytokine levels, such as in the evaluation of IL-6, where the levels were sometimes higher [86,88], sometimes comparable [83], and sometimes lower [85] for BMS patients than the controls. It remains debatable whether increased or decreased cytokine levels contribute to the etiopathogenesis of BMS [27].

When we focused on treatments, more specifically on LLLT, we found that the majority of the studies showed the effectiveness of laser therapy in reducing pain in BMS patients, although there is great diversity within the protocols for the laser parameters. The reduction in pain and burning sensations by LLLT has been attributed to various mechanisms. The analgesic effect seems to be due to the release of pain killers, such as beta-endorphins and enkephalins, and decreased secretion of pain mediators, such as bradykinin and histamine [134,135,136,137]. Pain relief is also attributed to a decrease in the activity of C fibres [21,31,36]. Nonetheless, the majority of studies on LLLT did not compare it with placebo groups, and the placebo effect of laser can be high.

For pharmacological treatment, as Silvestre et al. analysed in their review, drug treatment for the burning sensation in primary BMS of peripheral origin can consist of topical clonazepam, while central-type BMS appears to improve with the use of antidepressants, such as duloxetine, and antiseizure drugs, such as gabapentin [138].

### 4.2. Arguments for a Psychogenic Pathogenesis

The role of psychological factors in BMS has been the focus of several studies. From a historical point of view, the authors indirectly suggested a role of psychological factors in the aetiology of BMS by different proposals of the classification of BMS, such as Lamey et al. in 1996 who identified three types of BMS: type I, which is characterized by a burning sensation in the mouth and tongue during the day and is not linked to psychiatric disorders; type II, which is characterized by constant pain during the day and is linked to psychiatric disorders, particularly chronic anxiety; and type III, which is characterized by intermittent pain with atypical localization, such as in the cheek mucous membrane or tongue root, associated contact stomatitis, reactions to food additives, and the involvement of unspecified psychiatric disorders [139].

Most of the studies examined psychiatric disorders with a principal focus on anxiety and/or depression by using several different instruments/questionnaires. Other studies have focused on the personality characteristics of BMS patients, such as alexithymic traits or mental/personality disorders. Anxiety and depression seem to be the most common comorbid disorders among patients with BMS. Like many chronic pain disorders, BMS involves chronic pain with a late age onset (postmenopausal women) and a clear prevalence in women, both of which contribute to the debate regarding the aetiology of BMS because they imply the involvement of hormonal factors. While the majority of studies focused on current symptoms, some others examined the history of psychological symptoms [140] and found that the onset of major depression, generalized anxiety disorder, and painful conditions other than orofacial disorders preceded the onset of BMS in almost 80% of cases. [125]. Alexithymia, a personality trait involving dysregulation of negative affect [141], is a disorder that tends to be more prevalent in patients with chronic pain [142], as in BMS patients who are almost 80% alexithymic [114]. Several features of the personality of BMS patients are shared with other kinds of chronic pain, such as lower levels of self-directness and higher levels of harm avoidance than in the controls [117]. Other than anxiety and depression, a subset of psychological symptoms seems to contribute to the development and severity of BMS.

## 5. Conclusions

**Figure 2:**
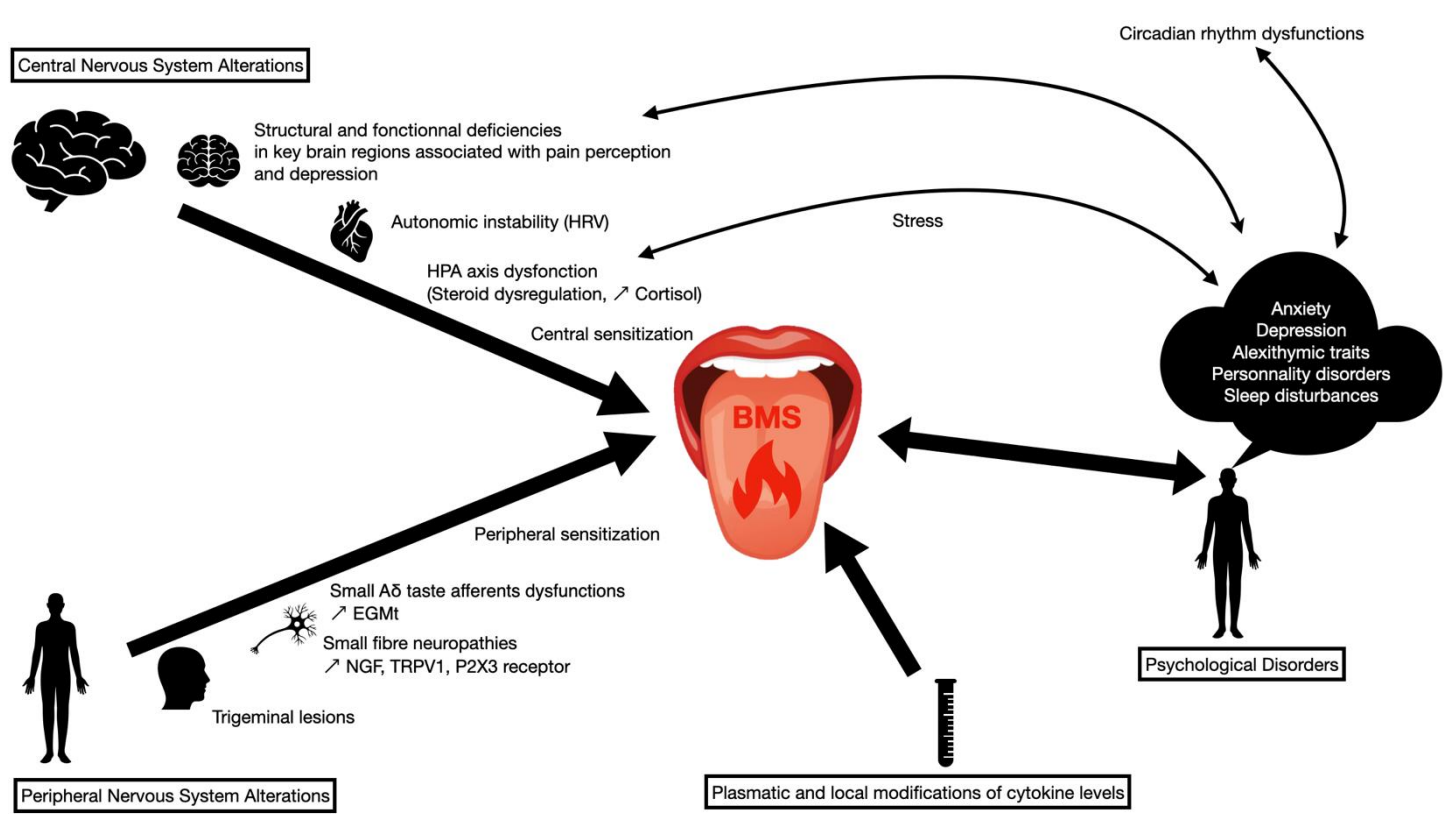
Pathophysiological hypotheses of burning mouth syndrome. HRV: heart rate variability; HPA axis: hypothalamic–pituitary–adrenal axis; EGMt: electrogustometric thresholds; NGF: nerve growth factor; TRPV1: transient receptor potential vanilloid type 1.

Both neurogenic and psychogenic components may be involved in the pathophysiology of BMS (Figure 2). The International Association for the Study of Pain (IASP) recently defined three different pains: nociceptive pain, “pain that arises from actual or threatened damage to non-neural tissue and is due to the activation of nociceptors”; neuropathic pain, “pain caused by a lesion or disease of the somatosensory nervous system”; and nociplastic pain, “pain that arises from altered nociception despite no clear evidence of actual or threatened tissue damage causing the activation of peripheral nociceptors and no evidence of a disease or lesion of the somatosensory system causing the pain” [143,144,145]. Hence, we can consider BMS as nociplastic pain. According to IASP, patients can have a combination of nociceptive pain that arises from actual or threatened damage to nonneural tissue and is due to the activation of nociceptors and nociplastic pain.

## Figures and Tables

**Table 1:** Cytokine evaluation of the study group.

Author/Year	Type of Study	Cytokine(s) Evaluated	Sample Type	Significant Results
Barry, 2018 [83]	Case control	IL-8, IL-4, IL-2, IL-12p70, IL-13, IFN-c, IL-6, IL-10, TNF–a, IL-1b	Plasma	IL-8 increased in BMS compared to healthy control IL-4, IL-2, IL-12p70, IL-13, IFN-c, IL-6, IL-10, TNF–a, and IL-1b comparable between the two groups
Ji, 2017 [84]	Case control	IL-18	Saliva	IL-18 increased in BMS compared to healthy control
Chen, 2007 [85]	Case control	IL-6	Blood	IL-6 is lower in BMS compared to healthy control
Treldal, 2020 [88]	RCT	IL-6, IL-8, IL-17A, IL-23 TNF-α	Blood Plasma Saliva	IL-6, IL-8, IL-17, IL-23, and TNF-α: higher in the No effect group than the patients in the Effect group, but not statistically significant (*p*-values: 0.068–0.916). Higher levels of IL-6, IL-8, and IL-23 for the No effect group when compared with the Control group (*p*-values: 0.096–0.542). Only IL-6 was significantly elevated in stimulated parotid saliva for the No effect group (*p* = 0.020). The cytokine levels in chewing-stimulated whole saliva did not differ between the patient groups
Miyauchi, 2019 [86]	Case control	Neuroinflammation-related molecules Cytokine	Plasma	IL-1β, IL-1 receptor antagonist, IL-6, macrophage inflammatory protein-1β, and platelet-derived growth factor-bb significantly higher in patients than in controls.
Guimarães, 2006 [87]	Case control	Genetic polymorphisms of IL-1 beta and 5-HTTLPR	Oral mucosa swabs	No statistical difference in 5-HTTLPR genotypes between the case and control groups (*p* = 0.60) Significant increase in the IL-1b high production genotype CT in BMS subjects (*p* = 0.005) = > Association between BMS and IL-1b high producer genotype.

## Data Availability

All data are available on demand.
